# Commensal microbe-derived acetate suppresses NAFLD/NASH development via hepatic FFAR2 signalling in mice

**DOI:** 10.1186/s40168-021-01125-7

**Published:** 2021-09-16

**Authors:** Ryo Aoki, Masayoshi Onuki, Koya Hattori, Masato Ito, Takahiro Yamada, Kohei Kamikado, Yun-Gi Kim, Nobuhiro Nakamoto, Ikuo Kimura, Julie M. Clarke, Takanori Kanai, Koji Hase

**Affiliations:** 1 Department of Gastroenterology, School of Medicine, Keio University, Tokyo, 160-8582 Japan; 2 Institute of Health Sciences, Ezaki Glico Co., Ltd., Osaka, 555-8502 Japan; 3 Division of Biochemistry, Graduate School of Pharmaceutical Science and Faculty of Pharmacy, Keio University, Tokyo, 105-8512 Japan; 4 Research Center for Drug Discovery, Faculty of Pharmacy, Keio University, Tokyo, 105-8512 Japan; 5 Department of Applied Biological Science, Graduate School of Agriculture, Tokyo University of Agriculture and Technology, Tokyo, 183-8509 Japan; 6 CSIRO Health and Biosecurity, Adelaide, South Australia 5000 Australia; 7 International Research and Development Centre for Mucosal Vaccines, The Institute of Medical Science, The University of Tokyo (IMSUT), Tokyo, 108-8639 Japan

**Keywords:** NASH, NAFLD, Short-chain fatty acids, Acetate, Prebiotics, Inulin, FFAR2, Blautia, Bacteroides

## Abstract

**Background:**

Non-alcoholic liver disease (NAFLD) is the hepatic manifestation of metabolic syndrome, and it can progress to non-alcoholic steatohepatitis (NASH). Alterations in the gut microbiome have been implicated in the development of NAFLD/NASH, although the underlying mechanisms remain unclear.

**Results:**

We found that the consumption of the prebiotic inulin markedly ameliorated the phenotype of NAFLD/NASH, including hepatic steatosis and fibrosis, in mice. Inulin consumption resulted in global changes in the gut microbiome, including concomitant enrichment of the genera *Bacteroides* and *Blautia*, and increased concentrations of short-chain fatty acids, particularly acetate, in the gut lumen and portal blood. The consumption of acetate-releasing resistant starch protected against NAFLD development. Colonisation by *Bacteroides acidifaciens* and *Blautia producta* in germ-free mice resulted in synergetic effects on acetate production from inulin. Furthermore, the absence of free fatty acid receptor 2 (FFAR2), an acetate receptor, abolished the protective effect of inulin, as indicated by the more severe liver hypertrophy, hypercholesterolaemia and inflammation. These effects can be attributed to an exacerbation of insulin resistance in the liver, but not in muscle or adipose tissue.

**Conclusion:**

These findings demonstrated that the commensal microbiome–acetate–FFAR2 molecular circuit improves insulin sensitivity in the liver and prevents the development of NAFLD/NASH.

Video abstract

**Supplementary Information:**

The online version contains supplementary material available at 10.1186/s40168-021-01125-7.

## Background

Non-alcoholic fatty liver disease (NAFLD) affects ≥ 25% of the general population in the USA and East Asia [1, 2]. NAFLD is strongly associated with metabolic syndrome, particularly obesity, diabetes and insulin resistance. NAFLD is also characterised by greater lipid (triglyceride and cholesterol) deposition in the liver, hepatomegaly and high serum alanine aminotransferase (ALT) activity. Up to 25% of patients with NAFLD develop a more serious form of the disease termed non-alcoholic steatohepatitis (NASH), which is characterised by steatosis, inflammation, hepatocyte ballooning and fibrosis [2]. Despite the high prevalence of NAFLD and the related public health concerns, current care for NAFLD/NASH is often limited to weight loss and exercise, which are often difficult to maintain [3].

In recent years, several lines of evidence have suggested that the gut microbiome represents a significant environmental factor contributing to NAFLD development and its progression to NASH. The gut microbiome of patients with NAFLD exhibits structural changes such as increased gram-negative bacteria and genetic pathways for lipopolysaccharide (LPS) biosynthesis [4–7]. LPS activated Toll-like receptors and caused hepatic inflammation in an experimental model of NASH [8]. Furthermore, ethanol production by intestinal microbes is higher in patients with NAFLD/NASH [3, 9, 10], and NASH has also been associated with changes in the profiles of the bile acids, 3-(4-hydroxyphenyl)lactate and phenylacetic acid [6, 7, 11, 12]. Gut microbe-derived imidazole propionate also impairs glucose tolerance and insulin resistance in hepatocytes [11]. These observations raise the possibility that the microbial alterations in the intestine may significantly contribute to the pathogenesis of NASH, and therefore, manipulation of the gut microbiome represents an emerging strategy for the prevention of NAFLD/NASH.

Several clinical studies revealed that prebiotic supplementation reduces hepatic lipogenesis and prevents the development of NAFLD in humans [13, 14], although the molecular mechanisms of the host–microbe interactions affecting the susceptibility to NASH remain to be fully determined. The gut microbiome uses prebiotics as fermentation sources to produce various metabolites, such as short-chain fatty acids (SCFAs). SCFAs, especially butyrate, are efficiently used by the colonic epithelium as energy sources [15]. SCFAs also enhance mucosal barrier function by increasing mucin and IgA production in the intestinal mucosa. Furthermore, *n*-butyrate facilitates regulatory T cell differentiation by inhibiting histone deacetylases, which prevents intestinal inflammation. In addition, acetate negatively regulates insulin signalling in adipocytes, thereby suppressing fat deposition [16]. These previous studies illustrated that the beneficial effects of probiotics on host immunity and metabolism are at least partially mediated by gut microbiome-derived metabolites.

In the present study, we aimed to identify the mechanism by which the gut microbiome regulates diet-induced NAFLD/NASH development. We illustrated that prebiotic administration causes a substantial increase in the amount of acetate reaching the liver, in which it plays a critical role in the prevention of NAFLD/NASH progression. We also demonstrated that the acetate–FFAR2 molecular circuit is responsible for modulating lipid metabolism and insulin sensitivity in the liver.

## Results

### Prebiotic consumption prevents diet-induced NAFLD/NASH development

**Fig. 1:**
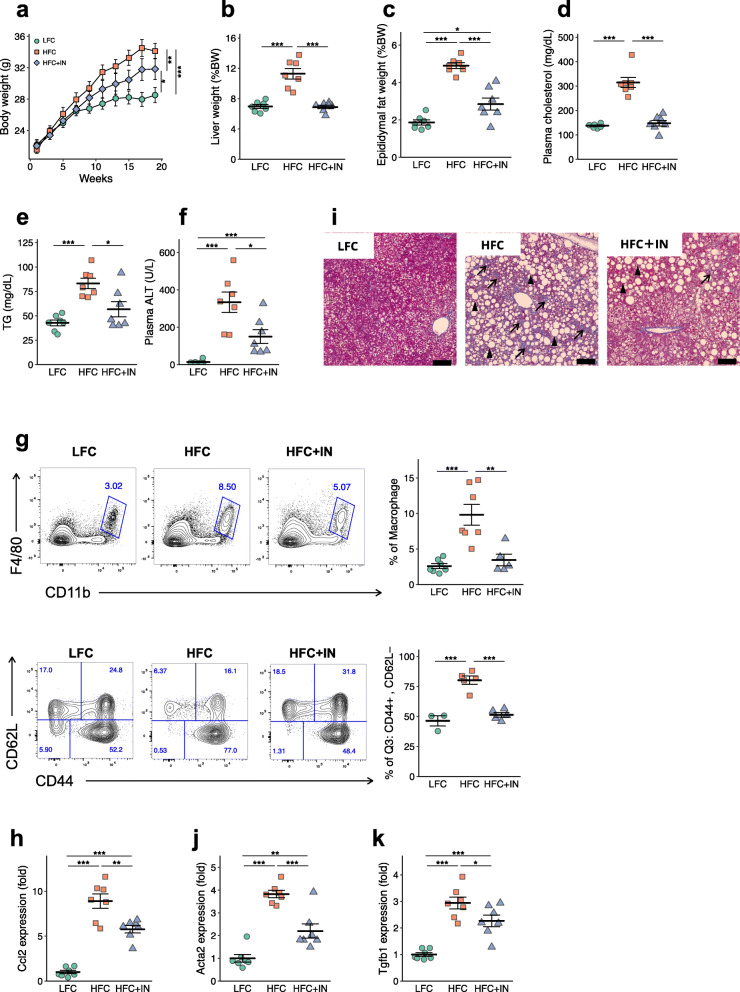
Prebiotic inulin supplementation prevents NAFLD/NASH development. Mice were fed a low-fat/fructose/cholesterol diet (LFC), a high-fat/fructose/cholesterol diet (HFC) or a 10% (w/w) inulin-supplemented HFC diet (HFC + IN) for 20 weeks. **a** Body mass of C57BL/6 mice (LFC, *n* = 8; HFC, *n* = 7; HFC + IN, *n* = 7). Data are mean ± SEM. **b** Liver to body mass ratio (LFC, *n* = 8; HFC, *n* = 7; HFC + IN, *n* = 7). **c** Epididymal fat to body mass ratio (LFC, *n* = 8; HFC, *n* = 7; HFC + IN, *n* = 7). **d** Plasma cholesterol. (LFC, *n* = 8; HFC, *n* = 7; HFC + IN, *n* = 7) **e** Plasma triglyceride (LFC, *n* = 8; HFC, *n* = 7; HFC + IN, *n* = 7). **f** Plasma ALT (LFC, *n* = 8; HFC, *n* = 7; HFC + IN, *n* = 7). **g** Representative plots and quantification of the flow cytometric analysis of liver mononuclear cells from LFD, HFC and HFC + IN-fed mice. CD11b^+^ and F4/80^+^ cells (LFC, *n* = 8; HFC, *n* = 7; HFC + IN, *n* = 7) and CD44^+^CD8^+^CD62L^−^ T cells (LFC, *n* = 3; HFC, *n* = 5; HFC + IN, *n* = 5) are shown. **i** Representative Masson’s trichrome staining of livers from LFC, HFC and HFC + IN mice. Scale bar: 50 μm; black arrows show fibrosis, black arrowheads show hepatocyte ballooning. **h**, **j**, **k** Quantitative PCR analysis of fibrosis-related genes in mRNA isolated from LFC, HFC and HFC + IN mouse livers(LFC, *n* = 8; HFC, *n* = 7; HFC + IN, *n* = 7). Each point in **b**–**f** and **h**–**m** represents an individual mouse (thick bars, means; error bars, SEM). The data represent at least two independent experiments with similar results. *P < 0.05, **P < 0.01, ***P < 0.001 (one-way analysis of variance (ANOVA) followed by post hoc Tukey’s test)

To determine the influence of the intestinal environment on the development of NAFLD/NASH, we fed mice either a low-fat/fructose/cholesterol (LFC), high-fat/fructose/cholesterol (HFC) or 10% (w/w) inulin-supplemented HFC diet (HFC+IN; Supplementary Table 1). Inulin has been reported to modulate the composition of the gut microbiome and facilitate intestinal microbial fermentation [17]. The consumption of the HFC diet created a phenotype typical of NAFLD, characterised by liver hypertrophy, hypercholesterolaemia and hypertriglyceridaemia in conjunction with obesity, compared with the effects of LFC diet feeding (Fig. 1a–e). Notably, the consumption of inulin almost completely abolished these pathological features. Serum ALT activity, a biological marker of hepatocellular damage, was also significantly lower in the HFC+IN group than in the HFC group (Fig. 1f), as was hepatic infiltration by monocytes/macrophages (CD11b^+^ F4/80^+^; Fig. 1g). These changes were accompanied by lower expression of *Ccl2*, which encodes a chemokine responsible for the recruitment of inflammatory monocytes, in the HFC+IN group (Fig. 1h). Furthermore, inulin consumption significantly reduced the number of liver-infiltrating CD8^+^ T cells with the effector/memory phenotype, which plays a role in the development of NASH [18] (Fig. 1g). Histological analysis illustrated that inulin consumption ameliorated hepatocyte ballooning and fibrosis (Fig. 1i and Supplementary Figure S1). Concomitantly, the expression of *Acta2* and *Tgfb1*, which encode α-smooth muscle actin and transforming growth factor-β, respectively, and mediate collagen deposition, was also significantly lower in the HFC+IN group than in the HFC group (Fig. 1j, k). Collectively, these data suggest that prebiotic inulin supplementation attenuates the development of HFC-induced NAFLD/NASH.

### Prebiotic supplementation causes global changes in the gut microbiome that result in increased SCFA production

**Fig. 2:**
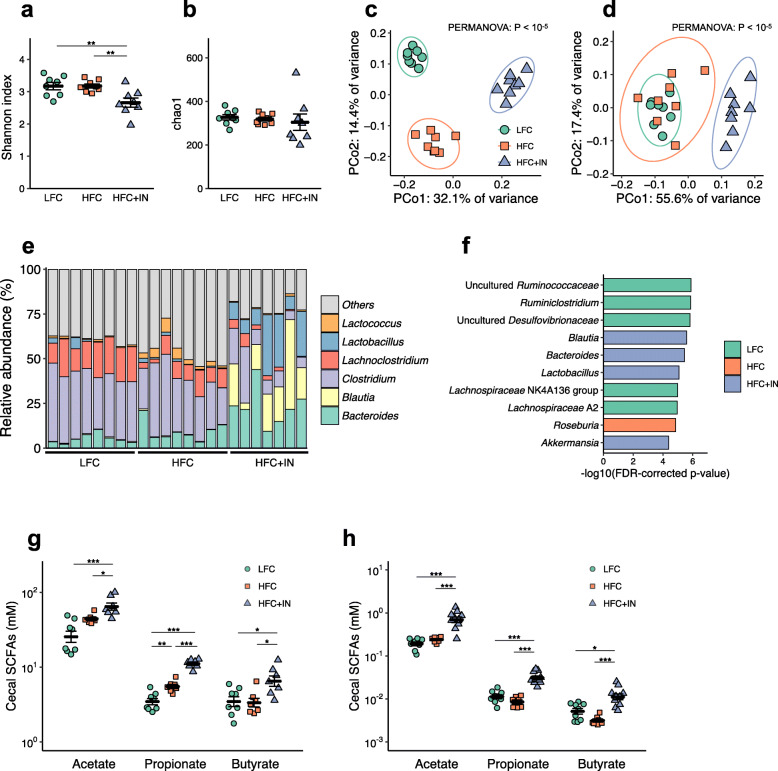
Inulin supplementation alters the composition of the gut microbiome and increases SCFA concentrations in the gut and portal blood. **a** Shannon index. **b** Chao1 index. **c** Principal coordinate analysis plot generated using an unweighted UniFrac metric. The two components explained 46.5% of the variance. **d** Principal coordinate analysis plot generated using a weighted UniFrac metric. The two components explained 73.0% of the variance. **e** Relative abundances of bacterial genera in the caecal contents. **f** Bacterial taxa at the genus level enriched in LFC, HFC or HFC+IN mice, generated using ALDEx2 analysis. Microbial taxa with the smallest FDR-corrected *p* values are shown. **g** SCFA concentrations in the caecum after 20 weeks of diet ingestion. **h** SCFA concentrations in the portal blood after 20 weeks of diet ingestion. Each point in **a**, **b**, **f** and **g** represents an individual mouse (thick bars, means; error bars, SEM). LFC, *n* = 8; HFC, *n* = 8; HFC + IN, *n* = 7. *P < 0.05, **P < 0.01, ***P < 0.001 on ANOVA followed by post hoc Tukey’s test (**a**, **b**, **g** and **h**)

We next investigated the impact of prebiotic consumption on the gut microbial community and its association with NAFLD/NASH development via 16S rRNA gene sequencing. We found that the numbers of Shannon index were significantly lower in LFC-fed mice (Fig. 2a), although there were no significant differences in the Chao1 index among the groups (Fig. 2b). Pielou’s evenness and taxonomic distinctness Λ^+^ were also decreased in LFC-IN-fed mice (Supplementary Figure S2). Principal coordinate analysis of the unweighted UniFrac metric profiled the three groups as independent clusters (Fig. 2c). However, weighted UniFrac analysis distinguished the HFC+IN-associated microbiome from those of the other two groups (Fig. 2d), indicating that the consumption of inulin induced considerable changes in the composition of the gut microbiome. Notably, bacterial differential abundance analysis using ALDEx2 demonstrated overrepresentation of the genera *Bacteroides*, *Blautia* and *Lactobacillus* in the HFC+IN-associated microbiome, with the lowest FDR-corrected *p* values (Fig. 2e, f and Supplementary Table 2).

This prebiotic-induced microbial alteration was likely to have affected the luminal metabolite profile. We next focused on the changes in SCFA levels because the dominant metabolites of *Bacteroides* and *Blautia* are SCFAs [19–22]. The dominant metabolite of *Lactobacillus* is lactate, which is eventually metabolised to other organic acids including SCFAs by gut microbes [23]. We found that the caecal and portal concentrations of all three SCFAs were significantly higher in the HFC+IN group than in the other two groups (Fig. 2g, h). Notably, acetate levels were considerably higher than propionate or butyrate levels in both caecal contents and portal blood. These findings suggested the possibility that SCFAs generated as prebiotic fermentation products, especially acetate, may play a key role in the prevention of NAFLD/NASH development.

### Luminal acetate protects against NAFLD

**Fig. 3:**
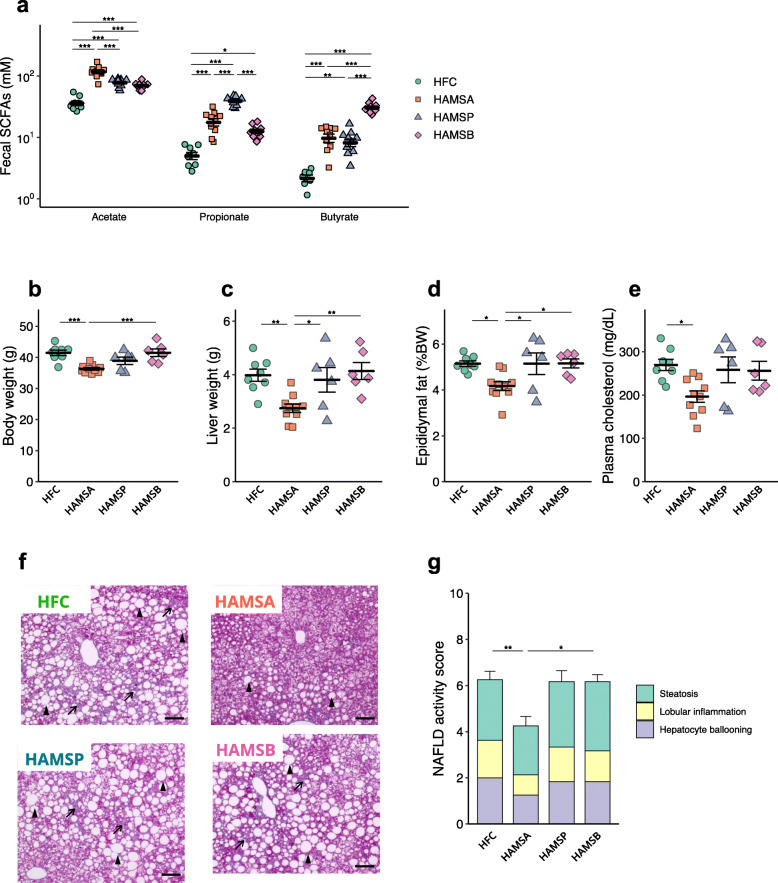
Acetate administered in the diet protects against NAFLD development. Mice were fed an HFC control diet (HFC), or HFC supplemented with high-amylose maize starch esterified with acetate (HAMSA), propionate (HAMSP) or butyrate (HAMSB) for 8 weeks (**a**) or 20 weeks (**b**–**g**). **a** Concentrations of acetate, propionate and butyrate in the faeces after 8 weeks consuming diets (HFC, *n* = 8; HAMSA, *n* = 9; HAMSP, *n* = 9; HAMSB = 9). **b** Body mass. **c** Liver to body mass ratio. **d** Epididymal adipose tissue to body mass ratio. **e** Quantification of plasma cholesterol. **f** Representative Masson’s trichrome staining of livers. Scale bar: 50 μm; black arrows show fibrosis, black arrowheads show hepatocyte ballooning. **g** Non-alcoholic fatty liver disease activity score. Each point in **a**–**e** represents an individual mouse (thick bars, means; error bars, SEM; **b**–**e** and **g**, HFC, *n* = 8; HAMSA, *n* = 9; HAMSP, *n* = 6; HAMSB = 6. Data are mean ± SEM. *P < 0.05, **P < 0.01, ***P < 0.001 on ANOVA followed by post hoc Tukey’s test (**a**–**e**) or Kruskal-Wallis followed by Steel-Dwass test (**g**)

To elucidate the effect of individual SCFAs on the development of NAFLD/NASH, we fed mice high-amylose maize starch (HAMS), a resistant starch, esterified with various types of SCFAs [24, 25]. We supplemented the HFC diet with acetylated HAMS (HAMSA), propionylated HAMS (HAMSP) or butyrylated HAMS (HAMSB) (Supplementary Table 1). Significantly higher levels of faecal acetate, propionate and butyrate were observed following supplementation with HAMSA, HAMSP and HAMSB, respectively (Fig. 3a). Of these, supplementation with HAMSA significantly ameliorated body mass gain, liver hypertrophy and epididymal fat deposition (Fig. 3b–d). HAMSA intake also ameliorated hypercholesterolaemia (Fig. 3e). Conversely, HAMSP and HAMSB failed to ameliorate any of these pathological changes induced by HFC feeding. Histological analysis also revealed that HAMSA, but not HAMSP and HAMSB, protected against NALFD symptoms (Fig. 3f, g). Thus, high intestinal acetate levels, but not propionate or butyrate levels, protected against HFC diet-induced liver pathology.

### Bacteroides acidifaciens and Blautia producta cooperatively produce acetate via the fermentation of inulin

**Fig. 4:**
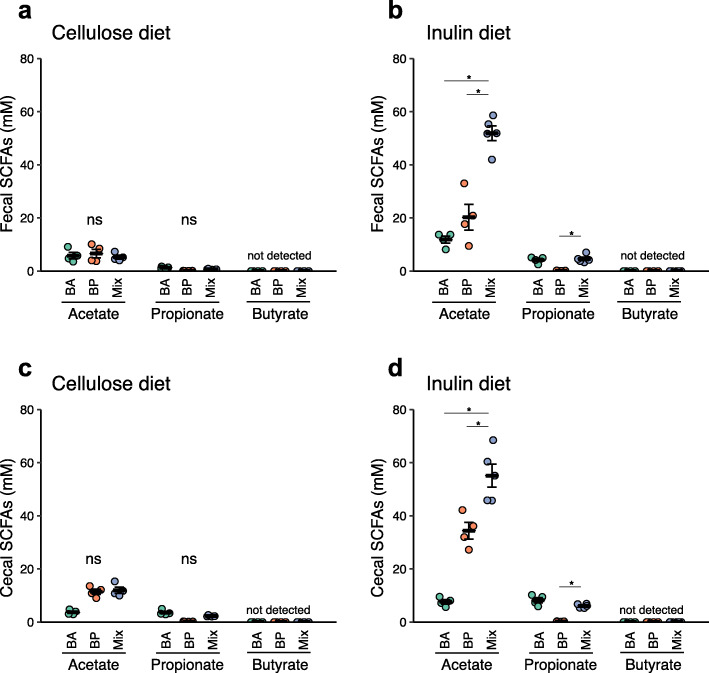
*Bacteroides acidifaciens* and *Blautia producta* produce acetate from the fermentation of inulin. **a**–**d** SCFA concentrations *Bacteroides acidifaciens* (BA), *Blautia producta* (BP), or BA/BP mixture (mix)-administered mice. The mice were fed a diet containing cellulose (**a**, **c**) or inulin (**b**, **d**) as a source of dietary fibre for 4 weeks. SCFA content of the faeces in mice (**a** BA, *n* = 4; BP, *n* = 4, mix, *n* = 4; **b** BA, *n* = 4; BP, *n* = 4, mix, *n* = 5). SCFA content of the caecal contents (**c** BA, *n* = 4; BP, *n* = 4, mix, *n* = 4; **d** BA, *n* = 4; BP, *n* = 4, mix, *n* = 5). Each point represents an individual mouse (thick bars, means; error bars, SEM). *P < 0.05, **P < 0.01, ***P < 0.001 (Kruskal-Wallis followed by Steel-Dwass test)

We finally sought to identify the commensal bacteria responsible for acetate production induced by prebiotic consumption. Among the bacterial genera overrepresented in the HFC+IN group, *Blautia* and *Bacteroides* have been reported to generate acetate [20–22]. Therefore, we isolated *Blautia* and *Bacteroides* from the caecal contents of HFC + IN mice. Most isolated strains were defined as *Bacteroides acidifaciens* (BA) and *Blautia producta* (BP) (Supplementary Figure S3). The 16S rRNA genes of these strains exhibited more than 99% identity with the type strains (data not shown). To investigate whether these bacterial species contributed to the production of acetate from inulin, we orally inoculating one or both strains into germ-free (GF) mice. These gnotobiotic mice were fed a diet containing inulin or non-fermentable cellulose as a control. In the cellulose diet groups, BA and/or BP colonisation failed to increase luminal acetate levels (Fig. 4a, c). In inulin diet-fed mice, mono-colonisation by BP significantly increased acetate levels *versus* those in the cellulose diet-fed groups, whereas BA only marginally enhanced acetate production (Fig. 4b, d). Notably, the administration of both BA and BP resulted in a marked increase in acetate production upon inulin consumption, suggesting that these two species synergistically generate acetate through the fermentation of inulin. In addition, BA-inoculated mice fed the inulin-containing diet exhibited low-level propionate production, but there was no synergistic effect when BP was co-inoculated. However, butyrate was not detected in any faecal samples. However, butyrate was not detected in any faecal samples. We therefore reasoned that prebiotic consumption increases the populations of BA and BP, which play a major role by producing acetate, but not other SCFAs.

### FFAR2 deficiency exacerbates NAFLD/NASH

**Fig. 5:**
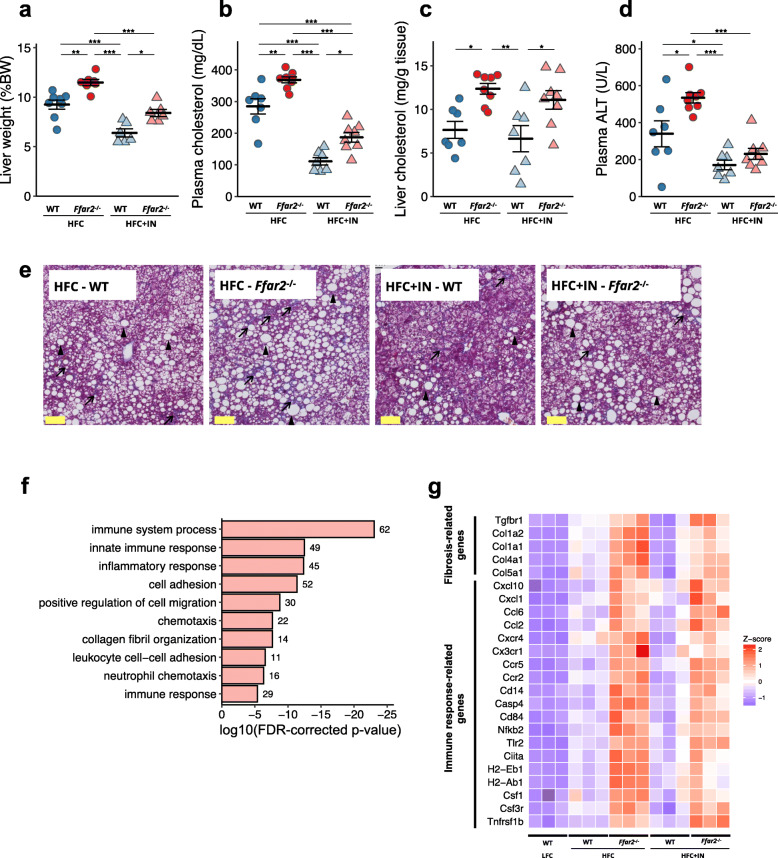
A deficiency in FFAR2 exacerbates the NAFLD/NASH phenotype. C57BL/6 WT or *Ffar2*^−/−^ mice were fed an HFC or an HFC + IN diet for 20 weeks. **a** Liver to body mass ratio (WT + HFC, *n* = 7; *Ffar2*^−/−^ + HFC, *n* = 8; WT + IN, *n* = 6, *Ffar2*^−/−^ + HFC, *n* = 6). **b** Plasma cholesterol (WT + HFC, *n* = 7; *Ffar2*^−/−^ + HFC, *n* = 8; WT + IN, *n* = 6, *Ffar2*^−/−^ + HFC, *n* = 6). **c** Liver cholesterol (WT + HFC, *n* = 7; *Ffar2*^−/−^ + HFC, *n* = 8; WT + IN, *n* = 7, *Ffar2*^−/−^ + HFC, *n* = 8). **d** Plasma ALT (WT + HFC, *n* = 7; *Ffar2*^−/−^ + HFC, *n* = 8; WT + IN, *n* = 7, *Ffar2*^−/−^ + HFC, *n* = 8). **e** Representative Masson’s trichrome staining of livers. Scale bar: 50 μm; black arrows show fibrosis, black arrowheads show hepatocyte ballooning. **f** Annotated gene ontology (GO) biological processes were assigned to genes upregulated in *Ffar2*^−/−^ mice versus WT mice in the liver after 20 weeks of consuming the HFC diet. Numbers next to bars represent the number of genes per pathway. **g** Heat maps of representative immune response- and fibrosis-related genes were constructed for genes differentially expressed in *Ffar2*^−/−^ mouse liver, as determined by RNA-seq. *n* = 3 per condition. Each point in **a**–**f** represents an individual mouse (thick bars, means; error bars, SEM). Data represent at least two independent experiments with similar results. *P < 0.05, **P < 0.01, ***P < 0.001 (ANOVA followed by post hoc Tukey’s test)

Acetate is well known to serve as a ligand for FFAR3 and FFAR2 [16, 26]. Therefore, we evaluated the contribution of the acetate–FFAR3/FFAR2 molecular circuit to the regulation of NAFLD/NASH development by feeding *Ffar3*^*−*/*−*^, *Ffar2*^*−*/*−*^ and wild-type (WT) mice HFC or HFC+IN for 20 weeks. The results illustrated that FFAR3 deficiency did not detract from the beneficial effect of inulin on NAFLD/NASH development (Supplementary Fig. S4a–d). By contrast, FFAR2 deficiency dampened the effect of inulin, as evidenced by significant increases in liver hypertrophy, plasma cholesterol levels and ALT activity (Fig. 5a–d). Lipid deposition and fibrosis in the liver were also worse in *Ffar2*^*−*/*−*^ mice than in WT mice (Fig. 5e and Supplementary Fig. S5a). Consistent with these observations, FFAR2 deficiency upregulated gene subsets associated with immune response/inflammation and collagen fibril organisation (Fig. 5f). The former included various chemokines; the chemokine receptors *Nfkb2* (encoding nuclear factor κB p100) and *Tnfrsf1b* (encoding tumour necrosis factor receptor 2); and the major histocompatibility class II molecule-encoding genes *H2-Eb1* and *H2-Ab1*. The latter group included the collagens *Col1a1*, *Col1a2*, *Col4a1* and *Col5a1* and *Tgfbr1* encoding transforming growth factor-β receptor 1 (Fig. 5g). Interestingly, FFAR2 deficiency did not exacerbate the obesity phenotype of the mice (Supplementary Fig. S5b–d). Taken together, these findings suggest that the acetate–FFAR2 molecular circuit protects against the development of NAFLD/NASH by directly regulating hepatic metabolism, rather than by ameliorating obesity.

### FFAR2 deficiency increases hepatic insulin resistance

**Fig. 6:**
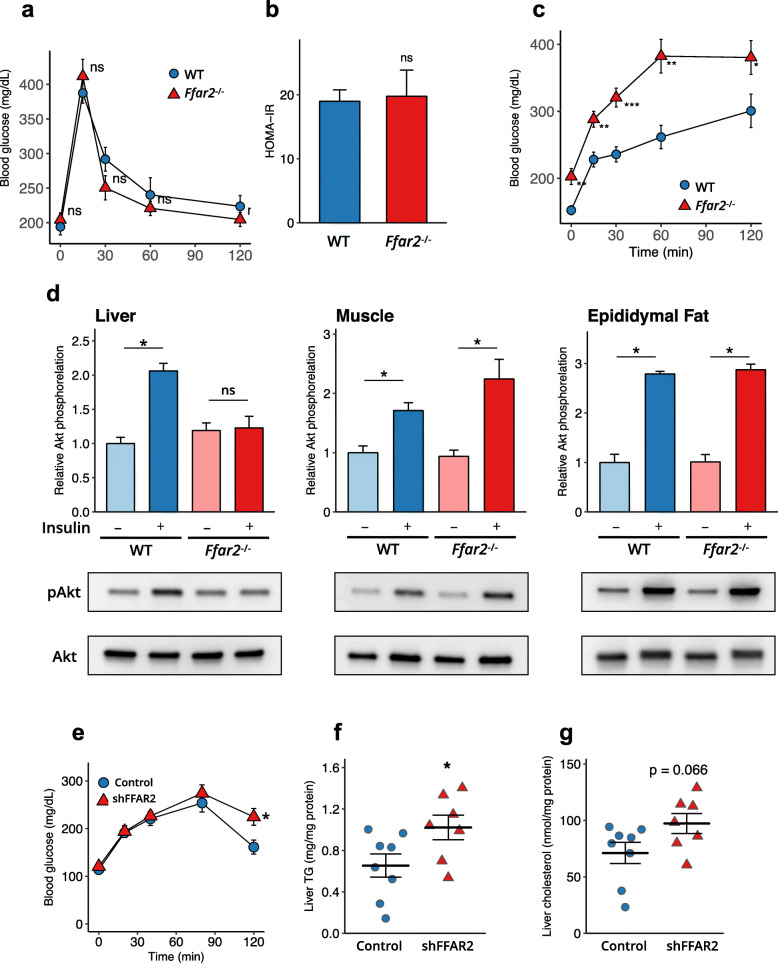
FFAR2 signalling play key roles in hepatic insulin signalling and NAFLD/NASH development. Impairment in the FFAR2-induced enhancement of insulin signalling exacerbates insulin resistance in the liver but not in the adipose tissue or muscle. **a** Oral glucose tolerance testing performed in WT and *Ffar2*^−/−^ mice fed an HFC diet for 19 weeks (WT, *n* = 6; *Ffar2*^−/−^, *n* = 7). **b** HOMA-IR was calculated according to the following formula: HOMA-IR = {[fasting insulin (mU/ml) − fasting glucose (mg/dl)]/405} (WT, *n* = 6; *Ffar2*^−/−^, *n* = 7). **c** Pyruvate tolerance testing performed in WT and *Ffar2*^*−*/−^ mice fed an HFC diet for 20 weeks (WT, *n* = 6; *Ffar2*^−/−^, *n* = 7). **d** Insulin-stimulated Akt phosphorylation at Ser_473_ in the liver, muscle and epididymal fat of *Ffar2*^−/−^ mice fed an HFC diet, after 6 h of fasting (*n* = 3 each). **e** Adenovirus-mediated *Ffar2* knockdown in the liver increases insulin resistance and triglyceride accumulation in the liver. Male 8-week-old C57BL/6 mice were administered with a control or *Ffar2* shRNA adenovirus (Control, *n* = 8; shFFAR2, *n* = 7) and were fed an HFC diet for 4 weeks. Pyruvate tolerance testing performed after 16 h of fasting. **f** Liver triglyceride. **g** Liver cholesterol. Data represent mean ± SEM. *P < 0.05, **P < 0.01, ***P < 0.001 (unpaired Student’s *t* test)

Consumption of a high-fat, high-fructose diet is known to induce insulin resistance in both muscle and the liver, which has been implicated in the development of NAFLD [27]. Therefore, we interrogated the effect of FFAR2 signalling on glucose metabolism and insulin sensitivity. In the oral glucose tolerance test, the blood glucose profiles of WT and *Ffar2*^−/−^ mice fed the HFC diet were similar (Fig. 6a), indicating that FFAR2 deficiency did not alter the severity of glucose intolerance. Likewise, the homeostasis model assessment of insulin resistance (HOMA-IR) was comparable between the two groups, indicative of similar levels of insulin resistance (Fig. 6b). In sharp contrast, pyruvate tolerance testing revealed that the severity of insulin resistance was greater in *Ffar2*^−/−^ mice than in WT mice (Fig. 6c).

To elucidate a possible causal relationship between FFAR2 and hepatic insulin signalling, we quantified Akt phosphorylation following insulin or saline administration. FFAR2 deficiency blunted the insulin-induced phosphorylation of Akt in the liver, but not in muscle or epididymal adipose tissue (Fig. 6d). These data support the notion that FFAR2 signalling prevents hepatic insulin resistance induced by HFC diet consumption.

### Liver-specific silencing of Ffar2 exacerbates local insulin resistance and lipid metabolism dysregulation

**Fig. 7:**
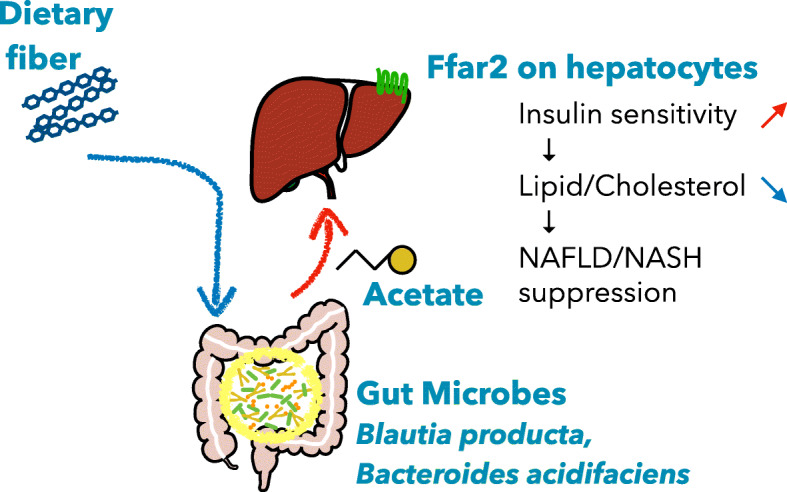
Proposed model for the protective effect of acetate derived from carbohydrate fermentation against NAFLD/NASH in mice

To further confirm the significance of hepatic FFAR2 in the regulation of hepatic insulin sensitivity and lipid metabolism, we performed in vivo silencing by administering an adenovirus encoding an siRNA targeting *Ffar2* (Ad-siFfar2). The adenoviral vector exhibits a preferential tropism for hepatocytes [28]. Two days after Ad-siFfar2 administration, *Ffar2* expression in the liver was decreased to 29.0 ± 0.8% of the level in the Ad-siCont–treated group. This liver-specific silencing of *Ffar2* exacerbated hepatic insulin resistance and lipid accumulation in HFC diet-fed mice (Fig. 6e–g). Taken together, these data imply that a prebiotic–acetate–FFAR2 molecular circuit prevents NAFLD/NASH progression by ameliorating hepatic insulin resistance and defects in lipid metabolism (Fig. 7).

## Discussion

In the present study, we revealed that an increase in luminal acetate levels following dietary supplementation with the prebiotic inulin or acetate-releasing resistant starch prevented the development of HFC diet-induced NAFLD/NASH. We also illustrated that FFAR2 deficiency exacerbated NAFLD/NASH progression and worsened insulin resistance in the liver. The consumption of the prebiotic resulted in the transportation of substantial acetate content to the liver via the portal vein, in which the SFCA activates hepatic FFAR2 signalling, thereby likely modulating hepatic insulin signalling to protect against steatosis.

The consumption of inulin had a major impact on the composition of the gut microbiome, including increases in the populations of BA and BP. Furthermore, we identified a synergistic effect of BA and BP colonisation on acetate production from inulin in the intestine, implying that there may be nutritional links between these two species. The genus *Bacteroides* is known to degrade a wide variety of complex carbohydrates [29], and several *Bacteroides* species can use inulin as a carbon source [30]. The genus *Blautia* is well known to produce acetate (it is an ‘acetogen’) via the Wood–Ljungdahl pathway, in addition to glycolysis [31]. Therefore, if large quantities of mono- or oligosaccharides, or other intermediate metabolites, are generated from the degradation of inulin by BA, these could be used by BP for further acetate production.

A recent clinical study revealed that *B. obeum*, formerly known as *Ruminococcus obeum*, was underrepresented in patients with NASH compared with its levels in patients with mild NAFLD, and a machine-learning model defined *B. obeum* as the most reliable biological marker for the differentiation of mild NAFLD from NASH. Furthermore, metagenomic analysis demonstrated that patients with NASH express lower levels of acetate-producing enzymes than those with mild NAFLD [4]. Considering these previous data and the present findings together, there is substantial evidence that prebiotics may be able to prevent the progression of NAFLD/NASH because they promote acetate production, at least partly depending on *Blautia* in humans.

We identified acetate, but not propionate or butyrate, as a key metabolite involved in the suppression of NAFLD/NASH development by administering resistant starches esterified with individual SCFAs. Acetate has been illustrated to be present at a much higher blood concentration (262 μM) than propionate (30 μM) or butyrate (30 μM) in humans [32]. Consistent with this finding, we additionally observed that the acetate concentration (760 μM) in portal blood surpassed those of propionate (32 μM) and butyrate (12 μM) in inulin-fed mice (Fig. 2h). Although all of the SCFAs are ligands of FFAR2, the EC_50_s of acetate, propionate and butyrate are in the low-millimolar range [33]. Thus, only acetate is considered able to activate FFAR2 signalling in the liver.

We also showed that deficiency of FFAR2, but not FFAR3, reduces the protective effect of inulin against NAFLD/NASH progression. FFAR2 deficiency exacerbated NAFLD/NASH without affecting the obese phenotype or peripheral glucose tolerance, which is not consistent with the results of a previous study, demonstrating that FFAR2 deficiency exacerbates obesity and glucose intolerance [16, 34]. We consider that this apparent discrepancy arose from differences in the composition of the diets used in the present and previous studies. Whereas the HFC diet used in this study contained high levels of fructose (20% of the total energy) and a relatively moderate amount of fat (30% of the total energy), to markedly upregulate lipogenesis in the liver, the previous study used a diet supplying 60% of its energy as fat to induce severe obesity and type 2 diabetes mellitus [16, 34]. Because prebiotic treatment improves hepatic insulin signalling only in the presence of FFAR2, it is likely that Ffar2 signalling specifically in the liver, rather than signalling in peripheral tissues, is essential for preventing NAFLD/NASH progression. In support of this view, liver-specific FFAR2 deficiency recapitulated the exacerbation of insulin resistance present in *Ffar2*^*−/−*^ mice.

Hepatic insulin resistance is closely associated with the development of NAFLD [35]. The impairment in hepatic insulin signalling causes *de novo* lipogenesis, which accelerates lipid deposition and the development of NAFLD [36]. Recent evidence underscored the significance of FFAR2 signalling in the regulation of glucose metabolism and insulin sensitivity in multiple tissues, such as the gut epithelium, adipose tissue and pancreatic β-cells. In addition, we propose in this study that hepatocyte Ffar2 signalling regulates insulin sensitivity and lipid metabolism. The activation of FFAR2 facilitates Akt phosphorylation upon stimulation with insulin; although the detailed pathway involved in FFAR2-mediated intracellular signalling remains to be established. Further studies are needed to clarify the role of FFAR2 signalling in the regulation of insulin signalling in the liver.

## Conclusion

Acetate derived from prebiotic fermentation in the gut lumen regulates hepatic lipid metabolism and insulin sensitivity via FFAR2 in hepatocytes, which prevents the progression of NAFLD/NASH (Fig. 7). Our findings provide evidence that the activation of hepatic FFAR2 by prebiotics or acetate-releasing resistant starch could represent a promising therapeutic strategy for NAFLD/NASH.

## Methods

### Mice and diet

Specific pathogen-free (SPF) C57BL/6 mice were purchased from SLC Inc. (Hamamatsu, Japan) and maintained under SPF conditions at the animal facility of Keio University. GF IQI mice were purchased from CLEA (Japan). GF and gnotobiotic mice were maintained in vinyl isolators, and fed with sterilised water and CMF diet (Oriental Yeast), unless otherwise mentioned. *Ffar2*^*−*/^ *−*[16] and *Ffar3*^*−*/*−*^ mice [26] were backcrossed against a C57BL/6 background. Male mice aged 8–12 weeks were used in all experiments and maintained under specific pathogen-free conditions. To induce NAFLD/NASH, a high-fat (40% kcal), high-fructose (22% w/w) and high-cholesterol (2% w/w) diet, containing trans fatty acids as the lipid source (HFC, Research Diets #D09100301), was provided. An LFC diet (10% kcal/fat) containing no fructose or cholesterol was used as the control diet (Research Diets #D09100304). The detailed composition of the LFC, HFC and HFC + IN diets is described in Supplementary Table 1. Inulin (10% w/w), HAMS (15% w/w), HAMSA (15% w/w), HAMSP (15% w/w) or HAMSB (15% w/w) was used to supplement the HFC diet in place of a similar quantity of cellulose (Supplementary Table 1). In the gnotobiotic mouse experiment, diets containing cellulose (AIN-93G, Research Diets #D10012G) or inulin as the dietary fibre source were used (Supplementary Table 3).

### Flow cytometric analysis of liver mononuclear cells

Liver mononuclear cells were isolated as described previously [37]. Briefly, livers were perfused via the portal vein, minced and passed through 100-μm nylon mesh. The filtrate was centrifuged at 50*×g* for 5 min to remove hepatocyte debris, and the supernatant was washed once. The cells were then resuspended in 40% Percoll (Thermo Fisher Scientific) and carefully overlaid onto 75% Percoll. The interface containing mononuclear cells was collected. After blocking with an anti-FcR antibody (CD16/32, BD) for 20 min at 4 °C, the mononuclear cells were incubated with specific fluorescence-labelled monoclonal antibodies at 4 °C for 30 min. The monoclonal antibodies used for cell identification are listed in Supplementary Table 4. The immunolabelled cells were identified using a FACS Canto II (BD), and the data were analysed using FlowJo software (Tree Star Inc.).

### Sequencing and processing of bacterial 16S rRNA genes in faecal DNA

Bacterial DNA was extracted from caecal contents as described previously [38]. The hypervariable V3–V4 region of the 16S gene was amplified using Ex Taq Hot Start (Takara Bio Inc.) with the primer set 5′-TCGTCGGCAGCGTCAGATGTGTATAAGAGACAGCCTACGGGNGGCWGCAG-3′ and 5′-GTCTCGTGGGCTCGGAGATGTGTATAAGAGACAGGACTACHVGGGTATCTAATCC-3′ and purified using AMPure XP (Beckman Coulter). Mixed samples were prepared by pooling approximately equal amounts of each amplified DNA species and sequenced using MiSeq Reagent Kit V3 (600 cycles) and a MiSeq sequencer (Illumina), in accordance with the manufacturer’s instructions. Sequences were analysed using QIIME version 1.9. Paired-end sequences were joined using the fastq-join tool in the ea-utils software package [39]. After trimming both primer sequences using cutadapt [40], following chimera detection using the USEARCH *de novo* method, the sequences were assigned to OTUs using the UCLUST algorithm with a sequence identity threshold of 97%. Taxonomic assignments for each OTU were made via similarity searching against the SILVA 16S rRNA gene sequence reference database (v132) [41] using blastn (v2.7.1) with default parameters [42]. The data were simplified to 10,000 sequences per sample using the rarefaction curves, and the relative abundances of the community members were determined using the rarefied data. To determine the bacterial taxonomy that explained the differences between the diet groups, ALDEx2 [43, 44] were used. β-diversity was estimated using the UniFrac metric to calculate the distances between the samples and visualised via principal coordinate analysis (PCoA). The result was statistically examined using permutational multivariate analysis of variance (PERMANOVA).

### Isolation of bacterial strains

The frozen stocks of caecal contents from mice fed the HFC + IN diet were diluted and anaerobically cultured on Gifu anaerobic medium (Nissui) or brain heart infusion (BHI, GE Healthcare) agar for 48–72 h in a chamber (Concept 400, Ruskin) gassed with a mixture of 80% nitrogen, 10% hydrogen and 10% carbon dioxide. The 16S rRNA genes of the isolated strains were sequenced using sequencing primers (27F, 518F, 800R and 1492R) [45, 46]. The primer sequences are listed in Supplementary Table 5. The sequences were aligned using Clustal Omega [47]. The 16S rRNA sequences of related species were aligned using PyNast and neighbour-joining phylogenetic tree was constructed using MEGA 6.0 [48].

### Gnotobiotic study design

BA and BP were anaerobically cultured in BHI broth for 16 h. Bacterial cells were collected via centrifugation (2000*×g*, 10 min) and then suspended with sterile saline. GF IQI mice (5–7 weeks old) were orally inoculated with 200 μL of the culture suspension of BA/BP alone or a mixture of BA/BP (approximately 1 × 10^9^ cfu) once. Two weeks after inoculation, the mice started consuming diets containing either cellulose or inulin as the dietary fibre source, and this continued for 4 weeks. Colonisation by BA and BP was microscopically monitored by examining the faeces of gnotobiotic mice.

### Oral glucose tolerance testing and HOMA-IR

Oral glucose tolerance testing was performed as follows. Mice were fasted for 6 h and then administered 2 g/kg glucose via gavage. The blood glucose concentration was determined using a glucose meter (OneTouch Ultra; Johnson & Johnson) and blood collected from the tail tip. In addition, plasma samples were collected before the glucose challenge to measure the insulin concentration using a commercial assay kit according to the manufacturer’s instructions (Mouse Insulin ELISA kit; Morinaga, Japan). HOMA-IR was calculated using the following formula: fasting insulin (μU/mL) × plasma glucose (mg/dL)/405.

### Pyruvate tolerance testing

Pyruvate tolerance testing was performed as follows. Mice were fasted for 16 h and then injected intraperitoneally with 1.5 g/kg sodium pyruvate (Sigma-Aldrich). The blood glucose concentration was determined using a glucose meter (OneTouch Ultra; Johnson & Johnson) and blood collected from the tail tip.

### Immunoblot analysis of insulin-stimulated Akt phosphorylation

After 20 weeks of HFC feeding, mice were fasted for 16 h. Saline or 0.75 mU/kg recombinant human insulin (Eli Lilly) was injected intraperitoneally. Fifteen minutes later, the liver, epididymal fat pads and skeletal muscle were quickly removed and homogenised in ice-cold lysis buffer (T-PER Tissue Protein Extraction Reagent [Thermo Fisher Scientific] containing protease inhibitor [Roche] and phosphatase inhibitor [Sigma Aldrich]). The homogenates were then snap-frozen in liquid nitrogen. Protein concentrations were determined using a BCA Protein Assay Kit (Thermo Fisher Scientific). Equal quantities of protein were loaded and resolved on polyacrylamide gels (7.5% Mini-Protean TGX gels, Bio-Rad), transferred to PVDF membranes (Bio-Rad) and blocked for 1 h with 5% BSA in PBS. Immunoblotting was performed using anti-pAkt (S473) (1:1000; D9E; 4060S; Cell Signalling) and anti-Akt1 primary antibodies (1:1000; c-20; sc-1618; Santa Cruz).

### Quantification of SCFA concentrations

Portal blood and caecal SCFA concentrations in inulin-fed mice were quantified via chromatography as described previously [49]. SCFA levels in the faeces and caecal contents of mice in the HAMS feeding experiments were quantified *via* gas chromatography–mass spectrometry using a modification of the method of Moreau et al. [50]. Approximately 50 mg of mouse caecal contents were homogenised in nine volumes of H_2_O (w/w). After centrifugation (10,000×*g* at 4 °C for 15 min), 200 μL of the supernatant were spiked with 10 μL of 1 mM 2-ethyl butyric acid as an internal standard and 20 μL of 20% (w/v) 5-sulfosalicylic acid solution for deproteinisation. After centrifugation (10,000×*g* at 4 °C for 15 min), 200 μL of the supernatant were acidified using 10 μL of 37% HCl, and organic acids were extracted via two rounds of extraction with 1 mL of diethyl ether. Then, 500 μL of the organic supernatant were mixed with 50 μL of *N*-tert-butyldimethylsilyl-*N*-methyltrifluoroacetamide (Sigma-Aldrich) in a new glass vial and left for 24 h at room temperature for derivatisation. The derivatised samples were passed through a JMS-Q1500GC GC/MS System (JEOL) equipped with an HP-5 capillary column (60 m × 0.25 mm × 0.25 μm, Agilent Technologies). Pure helium (99.9999%) was used a carrier gas and delivered at a flow rate of 1.2 mL/min. The following temperature programme was used: 60 °C (3 min), 60–120 °C (5 °C/min), 120–290 °C (20 °C/min) and 290 °C (3 min).

### RNA extraction and quantitative PCR (qPCR)

Tissues were excised, immediately submerged in RNAlater (Qiagen) and stored at 4 °C overnight and then at – 80 °C for subsequent RNA extraction. Total RNA was isolated from tissue samples using a QuickGene RNA tissue kit S II (Kurabo) and reverse-transcribed into cDNA using a High-Capacity cDNA Reverse Transcription Kit (Thermo Fisher Scientific). qPCR was performed using PrimeTime qPCR Primer/Probe Assays (Integrated DNA Technologies). The primers and probes used for qPCR are listed in Supplementary Table 6.

### RNA sequencing analysis

For RNA sequencing analysis, cDNA synthesis and library preparation were performed using a NEBNext Ultra RNA Library Prep Kit for Illumina (NEB) according to the manufacturer’s instructions. Libraries were sequenced using an Illumina HiSeq 2000 in the 50-bp single-end mode. The single-stranded sequenced reads were mapped to the mouse reference genome (mm10) and normalised to fragments per kilobase per million reads values using Tophat2 (v2.1.1) and Cufflinks (v2.2.0) software [51, 52]. Differential expression was determined by cuffdiff using an FDR cut-off of 0.05. Gene Ontology enrichment analysis was performed using the DAVID tool (v6.8) [53] on genes that with significantly differential expression.

### Biochemical and histologic analyses

The mice were sacrificed using deep isoflurane anaesthesia, and then blood and tissue samples were obtained. Plasma ALT activity and total cholesterol concentrations were measured using a Fuji Dri-Chem 3500 (Fujifilm). The hepatic lipid fraction was extracted from liver homogenates using the Bligh and Dyer method [54]. Hepatic cholesterol content was then measured using a Cholesterol Fluorometric Assay Kit (Cayman Chemicals), and hepatic triglyceride levels were measured using LabAssay Triglyceride (Wako). Liver tissues were fixed in 4% paraformaldehyde and embedded in paraffin. Sections (4 μm) were stained with H&E and a Masson trichrome solution. The NAFLD activity score was determined as described elsewhere [55, 56].

### Adenovirus-mediated RNA interference for Ffar2 in the mouse liver

Recombinant adenoviruses expressing mouse siFfar2 or control siRNA were purchased from Vector Biolabs. The viruses were titred using an Adeno-Rapid Titer Kit (Clontech) and administered via tail vein injection (1 × 10^9^ pfu virus per mouse). Four weeks after the injection, pyruvate tolerance testing was performed, and then the mice were sacrificed to collect their livers for analysis.

### Statistical analysis

Results are stated as the mean ± SEM. Histopathological scores and gnotobiotic experimental data were analysed using the non-parametric Kruskal-Wallis test to assess whether any differences occurred, followed by Steel-Dwass. All pairs test was used to assess the pairwise differences among the groups. The other data were tested for normality using Shapiro–Wilk test. Non-normally distributed data were log-transformed to normalise the distribution. Differences between two groups were analysed using unpaired Student’s *t* tests. Comparisons of more than two groups were performed using one-way ANOVA followed by Tukey’s multiple comparisons test. Results were considered statistically significant when *P* < 0.05, with the significance level indicated as ^*^P < 0.05, ^**^P < 0.01 and ^***^P < 0.001. Statistical analyses were conducted using R, version 3.6.3 (http://cran.r-project/org). Exact *P* value of statistical test and effect size are shown in the Supplementary Tables 7, 8, 9, 10, 11, 12, 13, 14 and 15.

## Supplementary Information

**Additional file 1: Supplemental Figure S1.** Non-alcoholic fatty liver disease activity score. Prebiotic inulin supplementation prevents NAFLD/NASH development. Mice were fed a low-fat/fructose/cholesterol diet (LFC), a high-fat/fructose/cholesterol diet (HFC) or a 10% (w/w) inulin-supplemented HFC diet (HFC+IN) for 20 weeks. Data are mean ± SEM. *P<0.05, **P<0.01, ***P<0.001 (Kruskal-Wallis followed by Steel-Dwass test). **Supplemental Figure S2.** Alpha diversity index. a, Pielou evenness. b, Simpson reciprocal index. c, Taxonomic distinctness Λ+. Each point represents an individual mouse (thick bars, means; error bars, SEM). *P<0.05, **P<0.01, ***P<0.001 (ANOVA followed by *post hoc* Tukey’s test). **Supplemental Figure S3.** Phylogenetic tree predicted by the neighbour-joining method using 16S rRNA gene sequences a, Isolated strains 160, 169 and 174 belong to the Bacteroides acidifaciens cluster. Strain 174 was used for the gnotobiotic experiment. b, Isolated strains 1, 2 and 5 belong to the Blautia producta cluster. Strain 1 was used for the gnotobiotic experiment. Bootstrap values are expressed as percentages of 1000 replications. The scale bars show evolutionary distances in units of the number of nucleotide substitutions per site. **Supplemental Figure S4.** A deficiency of Ffar3 does not affect the features of NAFLD/NASH C57BL/6 WT and *Ffar3*−/− mice were fed an HFC or an HFC+IN diet for 20 weeks. a, Body mass. b, Liver to body mass ratio. c, Plasma cholesterol. d, Plasma ALT. Each point represents an individual mouse (thick bars, means; error bars, SEM).*P<0.05, **P<0.01, ***P<0.001 (ANOVA followed by *post hoc* Tukey’s test). **Supplemental Figure S5.** a deficiency in Ffar2 does not affect the obese phenotype but NAFLD. a, non-alcoholic fatty liver disease activity score. b, Body mass. c, Epididymal fat to body mass ratio. c, Plasma triglyceride. Each point represents an individual mouse (thick bars, means; error bars, SEM). Data represent at least two independent experiments with similar results. Data are mean ± SEM. *P<0.05, **P<0.01, ***P<0.001 on Kruskal-Wallis followed by Steel-Dwass test (a) or ANOVA followed by *post hoc* Tukey’s test (b-d).

**Additional file 2: Supplementary Table 1.** Composition of diets used in the experiment. **Supplementary Table 2.** Summary of differential abundance. Most abundant group in three groups were highlighted. **Supplementary Table 3.** Composition of diets used in the experiment. **Supplementary Table 4.** Antibodies used for flow cytometry. **Supplementary Table 5.** Probes used for quantitative real-time PCR. **Supplementary Table 6.** Primers of 16S rRNA and sequence used in the present study.

**Additional file 3: Supplementary Table 7.** Summary of statistical analysis. Exact p-values of test estimates and effect size (related to Fig. 1). **Supplementary Table 8.** Summary of statistical analysis. Exact *p*-values of test estimates and effect size (related to Fig. 2). **Supplementary Table 9.** Summary of statistical analysis. Exact *p*-values of test estimates and effect size (related to Fig. 3). **Supplementary Table 10.** Summary of statistical analysis. Exact *p*-values of test estimates and effect size (related to Fig. 4). **Supplementary Table 11.** Summary of statistical analysis. Exact *p*-values of test estimates and effect size (related to Fig. 5). **Supplementary Table 12.** Summary of statistical analysis. Exact *p*-values of test estimates and effect size (related to Fig. 6). **Supplementary Table 13.** Summary of statistical analysis. Exact *p*-values of test estimates and effect size

## Data Availability

The data supporting the findings of this study are available from the corresponding author upon request. The sequencing data have been deposited in the DNA Data Bank of Japan (DDBJ), under the accession numbers PRJDB8684 for 16S rRNA sequencing data and PRJDB8686 for RNA sequencing data. 16S rRNA gene sequences of isolated BA and BP have been deposited in DDBJ (the accession numbers LC635509-LC635514).

## Abbreviations

NAFLD: Non-alcoholic liver disease
NASH: Non-alcoholic steatohepatitis
ALT: Alanine aminotransferase
LPS: Lipopolysaccharide
LFC: Low-fat/fructose/cholesterol
HFC: High-fat/fructose/cholesterol
OTUs: Operational taxonomic units
HAMS: High-amylose maize starch
HAMSA: Acetylated HAMS
HAMSP: Propionylated HAMS
HAMSB: Butyrylated HAMS
BA: *Bacteroides acidifaciens*
BP: *Blautia producta*
