# Supplementary material for: Commensal microbe-derived acetate suppresses NAFLD/NASH development via hepatic FFAR2 signalling in mice
Source: Microbiome. 2021 Sep 16;9:188. doi: 10.1186/s40168-021-01125-7 (PMC8447789; doi:10.1186/s40168-021-01125-7)
Supplement: Supplementary file 2 — Additional file 1: Supplemental Figure S1. Non-alcoholic fatty liver disease activity score. Prebiotic inulin supplementation prevents NAFLD/NASH development. Mice were fed a low-fat/fructose/cholesterol diet (LFC), a high-fat/fructose/cholesterol diet (HFC) or a 10% (w/w) inulin-supplemented HFC diet (HFC+IN) for 20 weeks. Data are mean ± SEM. *P<0.05, **P<0.01, ***P<0.001 (Kruskal-Wallis followed by Steel-Dwass test). Supplemental Figure S2. Alpha diversity index. a, Pielou evenness. b, Simpson reciprocal index. c, Taxonomic distinctness Λ+. Each point represents an individual mouse (thick bars, means; error bars, SEM). *P<0.05, **P<0.01, ***P<0.001 (ANOVA followed by post hoc Tukey’s test). Supplemental Figure S3. Phylogenetic tree predicted by the neighbour-joining method using 16S rRNA gene sequences a, Isolated strains 160, 169 and 174 belong to the Bacteroides acidifaciens cluster. Strain 174 was used for the gnotobiotic experiment. b, Isolated strains 1, 2 and 5 belong to the Blautia producta cluster. Strain 1 was used for the gnotobiotic experiment. Bootstrap values are expressed as percentages of 1000 replications. The scale bars show evolutionary distances in units of the number of nucleotide substitutions per site. Supplemental Figure S4. A deficiency of Ffar3 does not affect the features of NAFLD/NASH C57BL/6 WT and Ffar3−/− mice were fed an HFC or an HFC+IN diet for 20 weeks. a, Body mass. b, Liver to body mass ratio. c, Plasma cholesterol. d, Plasma ALT. Each point represents an individual mouse (thick bars, means; error bars, SEM).*P<0.05, **P<0.01, ***P<0.001 (ANOVA followed by post hoc Tukey’s test). Supplemental Figure S5. a deficiency in Ffar2 does not affect the obese phenotype but NAFLD. a, non-alcoholic fatty liver disease activity score. b, Body mass. c, Epididymal fat to body mass ratio. c, Plasma triglyceride. Each point represents an individual mouse (thick bars, means; error bars, SEM). Data represent at least two independent experiments [file 40168_2021_1125_MOESM2_ESM.pdf]

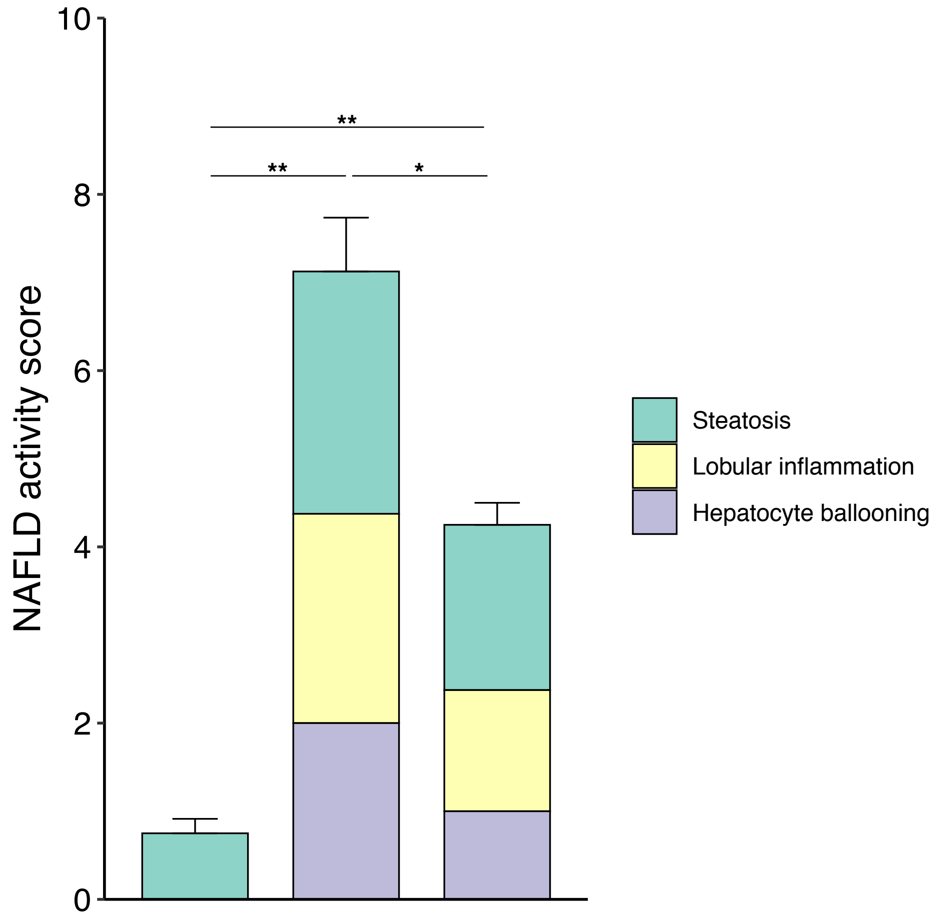

Supplemental Fig. S1: non-alcoholic fatty liver disease activity score
Prebiotic inulin supplementation prevents NAFLD/NASH development. Mice were fed a low-fat/fructose/cholesterol diet (LFC), a high-fat/fructose/cholesterol diet (HFC) or a 10% (w/w) inulin-supplemented HFC diet (HFC+IN) for 20 weeks. Data are mean ± SEM. *P<0.05, **P<0.01, ***P<0.001 (Kruskal-Wallis followed by Steel-Dwass test).

Supplementary Figure S1

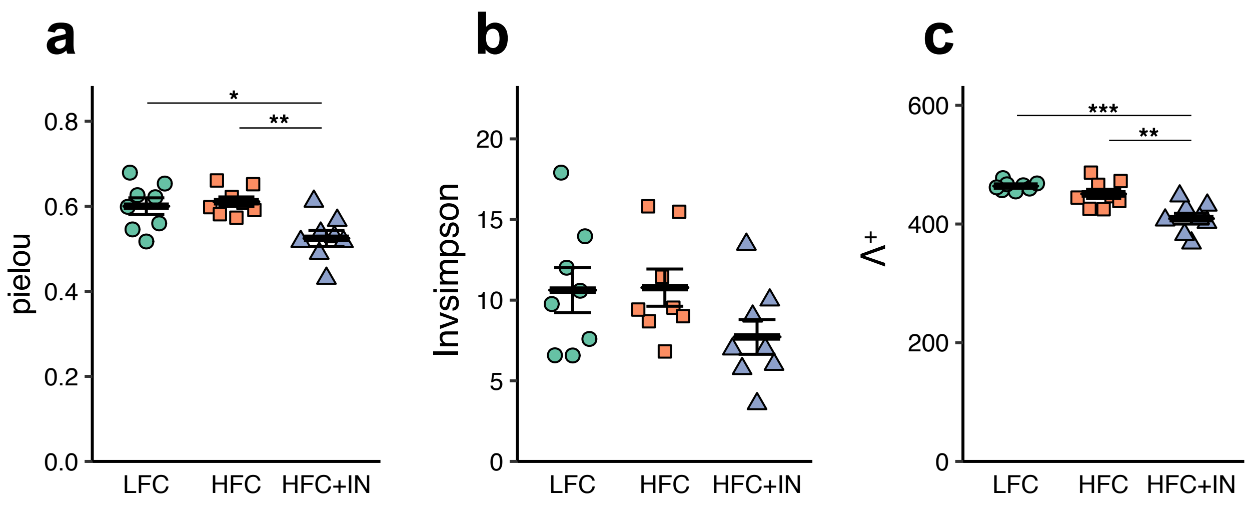

Supplemental Fig. S2: Alpha diversity index.
a, Pielou evenness. **b**, Simpson reciprocal index. **c**, Taxonomic distinctness Λ^+.
Each point represents an individual mouse (thick bars, means; error bars, SEM).
*P<0.05, **P<0.01, ***P<0.001 (ANOVA followed by *post hoc* Tukey's test).

Supplementary Figure S2

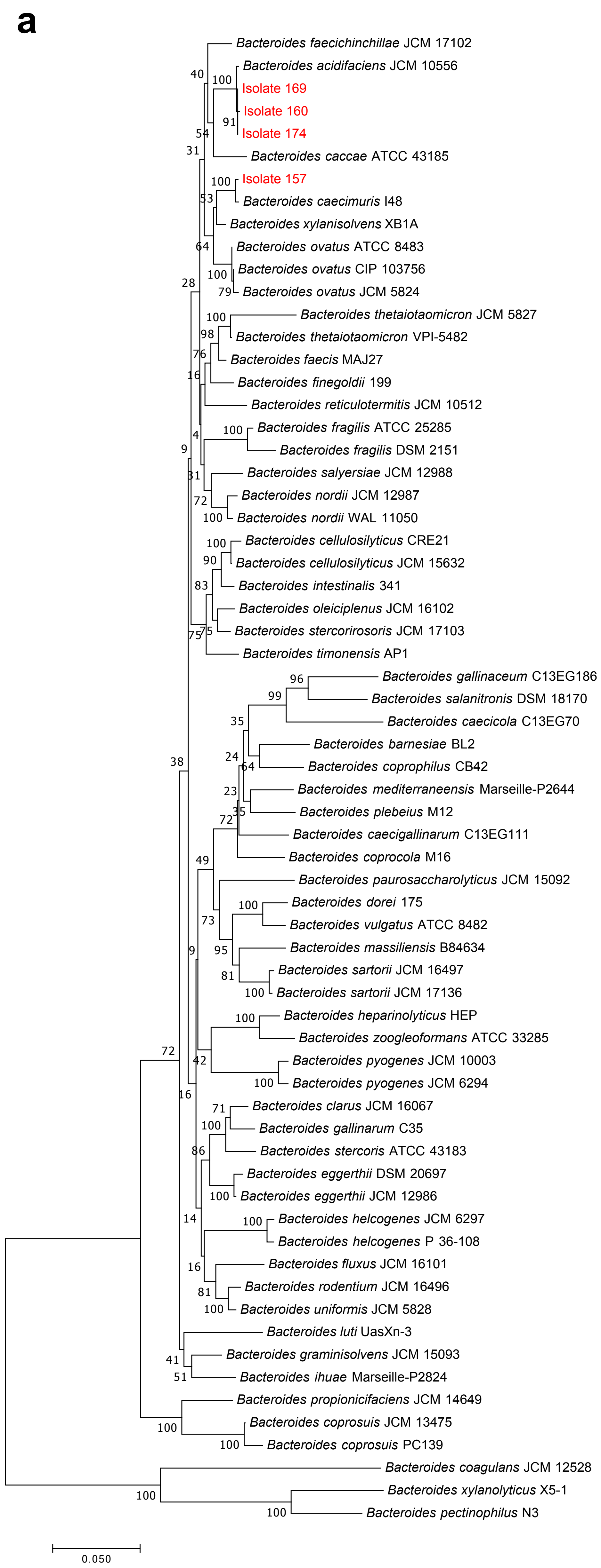

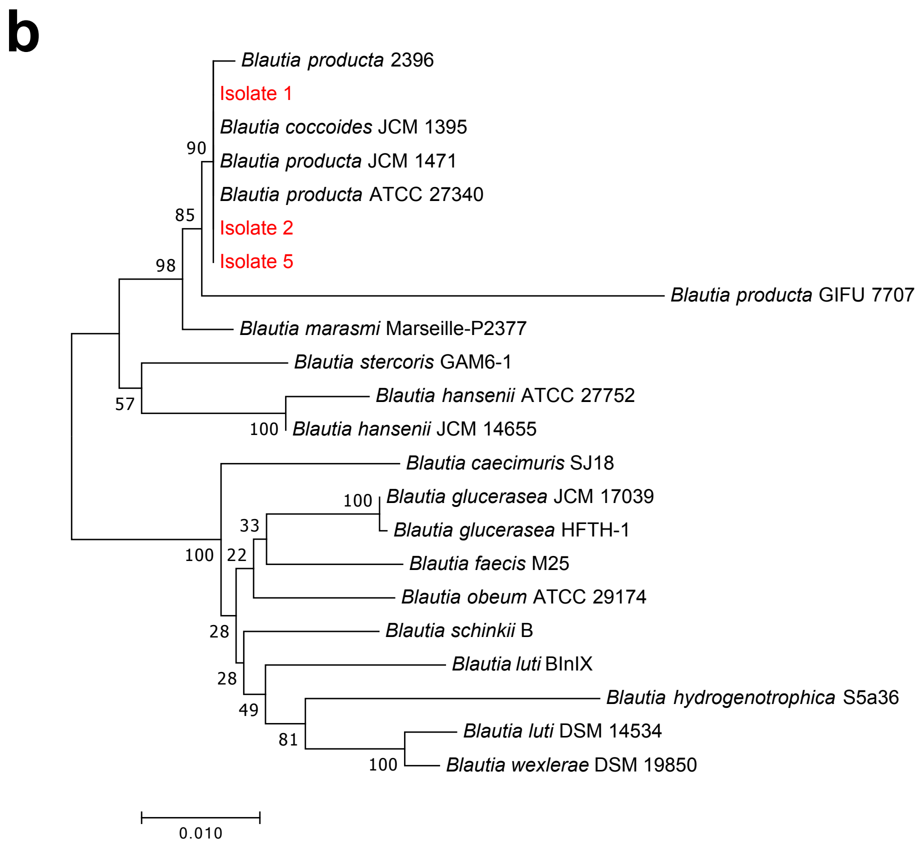

Supplemental Fig. S3: Phylogenetic tree predicted by the neighbour-joining method using 16S rRNA gene sequences
a, Isolated strains 160, 169 and 174 belong to the *Bacteroides acidifaciens* cluster. Strain 174 was used for the gnotobiotic experiment. **b**, Isolated strains 1, 2 and 5 belong to the *Blautia producta* cluster. Strain 1 was used for the gnotobiotic experiment. Bootstrap values are expressed as percentages of 1000 replications. The scale bars show evolutionary distances in units of the number of nucleotide substitutions per site.

Supplementary Figure S3

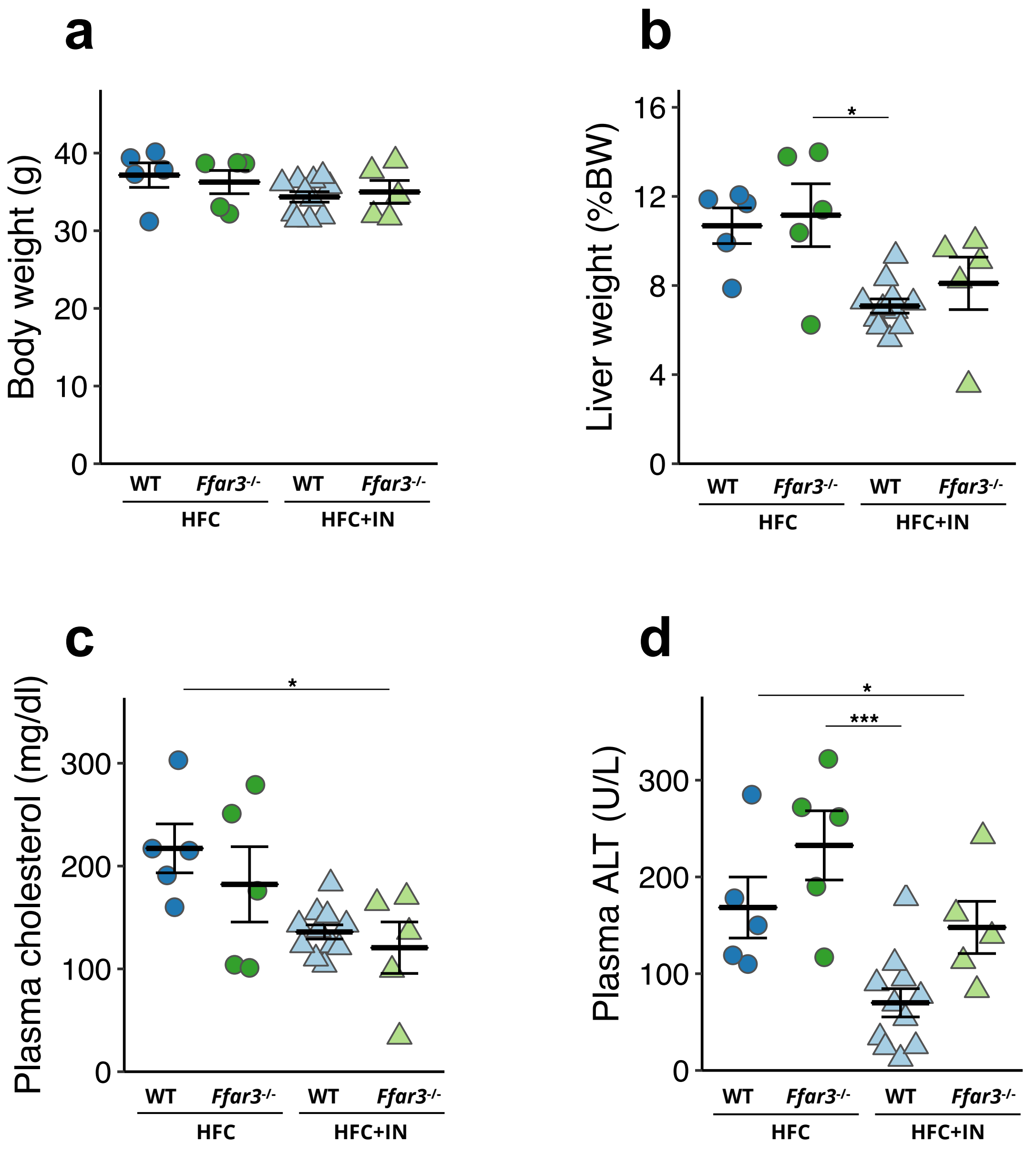

Supplemental Fig. S4: A deficiency of Ffar3 does not affect the features of NAFLD/NASH
C57BL/6 WT and *Ffar3*$^{-/-}$ mice were fed an HFC or an HFC+IN diet for 20 weeks. **a**, Body mass. **b**, Liver to body mass ratio. **c**, Plasma cholesterol. **d**, Plasma ALT. Each point represents an individual mouse (thick bars, means; error bars, SEM).*P<0.05, **P<0.01, ***P<0.001 (ANOVA followed by *post hoc* Tukey's test).

Supplementary Figure S4

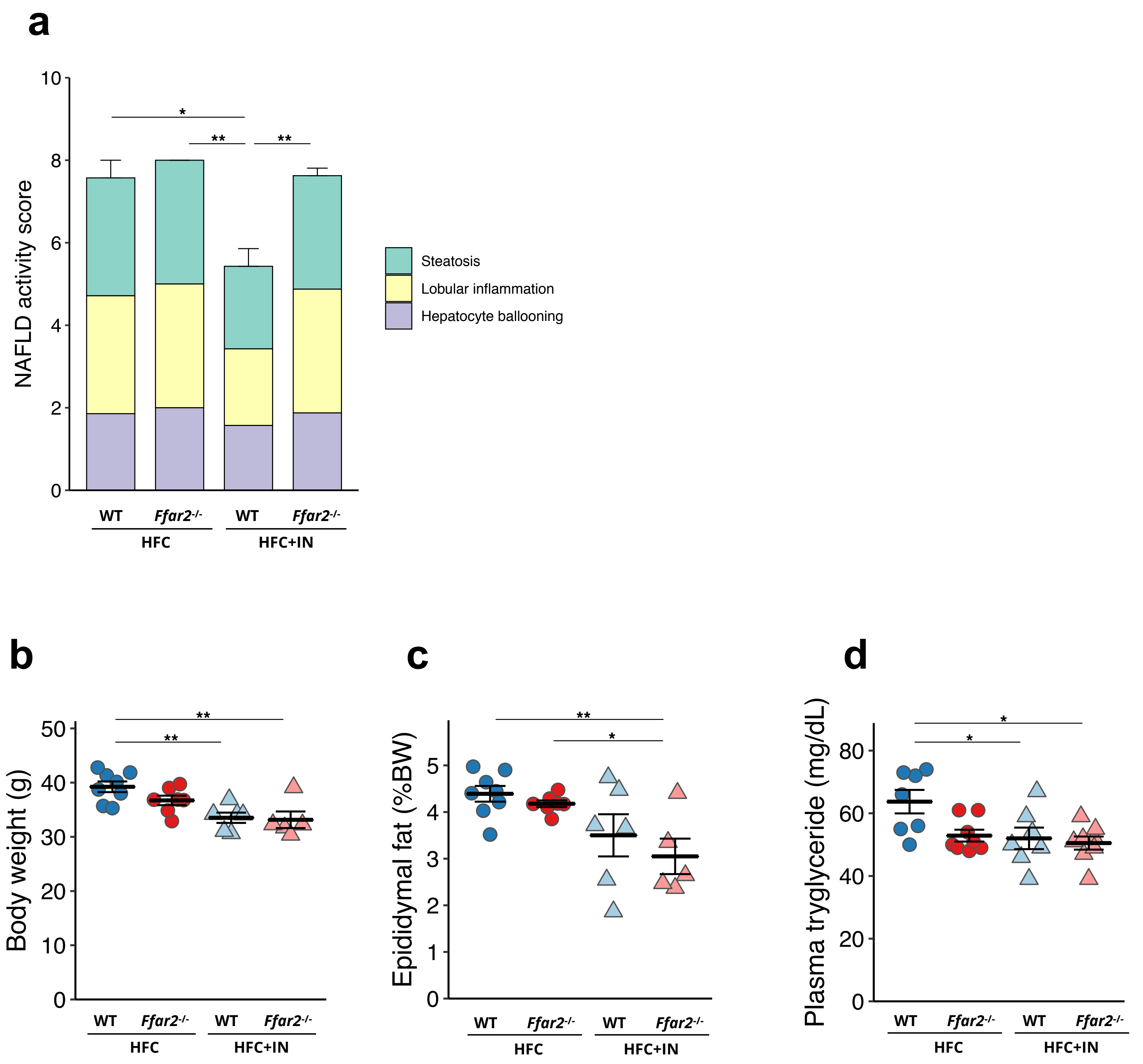

Supplemental Fig. S5: a deficiency in Ffar2 does not affect the obese phenotype but NAFLD.
a, non-alcoholic fatty liver disease activity score. **b**, Body mass. **c**, Epididymal fat to body mass ratio. **c**, Plasma triglyceride. Each point represents an individual mouse (thick bars, means; error bars, SEM). Data represent at least two independent experiments with similar results. Data are mean ± SEM. *P<0.05, **P<0.01, ***P<0.001 on Kruskal-Wallis followed by Steel-Dwass test (a) or ANOVA followed by *post hoc* Tukey's test (b-d).

Supplementary Figure S5
